# Mechanism of Action and Therapeutic Implications of Nrf2/HO-1 in Inflammatory Bowel Disease

**DOI:** 10.3390/antiox13081012

**Published:** 2024-08-20

**Authors:** Lingling Yuan, Yingyi Wang, Na Li, Xuli Yang, Xuhui Sun, Huai’e Tian, Yi Zhang

**Affiliations:** 1 Department of Gastroenterology, Hospital of Chengdu University of Traditional Chinese Medicine, Chengdu 610072, China; yuanlingling2022@stu.cdutcm.edu.cn (L.Y.); wangyingyi@stu.cdutcm.edu.cn (Y.W.); yxuli2023@stu.cdutcm.edu.cn (X.Y.); sxh261111@stu.cdutcm.edu.cn (X.S.); tiane@stu.cdutcm.edu.cn (H.T.); 2 Department of Infection, Hospital of Chengdu University of Traditional Chinese Medicine, Chengdu 610072, China; lina@stu.cdutcm.edu.cn

**Keywords:** Nrf2/HO-1, inflammatory bowel disease, oxidative stress, colorectal cancer

## Abstract

Oxidative stress (OS) is a key factor in the generation of various pathophysiological conditions. Nuclear factor erythroid 2 (NF-E2)-related factor 2 (Nrf2) is a major transcriptional regulator of antioxidant reactions. Heme oxygenase-1 (HO-1), a gene regulated by Nrf2, is one of the most critical cytoprotective molecules. In recent years, Nrf2/HO-1 has received widespread attention as a major regulatory pathway for intracellular defense against oxidative stress. It is considered as a potential target for the treatment of inflammatory bowel disease (IBD). This review highlights the mechanism of action and therapeutic significance of Nrf2/HO-1 in IBD and IBD complications (intestinal fibrosis and colorectal cancer (CRC)), as well as the potential of phytochemicals targeting Nrf2/HO-1 in the treatment of IBD. The results suggest that the therapeutic effects of Nrf2/HO-1 on IBD mainly involve the following aspects: (1) Controlling of oxidative stress to reduce intestinal inflammation and injury; (2) Regulation of intestinal flora to repair the intestinal mucosal barrier; and (3) Prevention of ferroptosis in intestinal epithelial cells. However, due to the complex role of Nrf2/HO-1, a more nuanced understanding of the exact mechanisms involved in Nrf2/HO-1 is the way forward for the treatment of IBD in the future.

## 1. Introduction

Inflammatory bowel disease (IBD) is a chronic relapsing inflammatory disease of the intestines, including ulcerative colitis (UC) and Crohn’s disease (CD), which is most often diagnosed in adolescents and youths [1]. Real-world data emphasize the increasing incidence and prevalence of IBD, a major global health problem that affects millions of people worldwide [2]. The burden of IBD remains high in countries with a high and medium–high socio-demographic index (SDI) and, in addition, an increasing burden is borne by countries with low and medium SDIs [3]. The etiology and mechanism of IBD have not been fully clarified to date. It is now internationally recognized that the pathogenesis mainly involves immune abnormalities, genetic factors, infectious factors, intestinal microbiota, and environmental factors [4]. Classical strategies for treating IBD include aminosalicylates, corticosteroids, immunosuppressants, and biotherapy, focusing on reducing intestinal mucosal inflammation, inducing and prolonging disease remission and managing complications [5]. However, these treatments usually have serious drawbacks, such as the inability to maintain the treatment to produce drug resistance and large adverse reactions, which cause problems for clinical treatment [6]. Therefore, people need more and more new diagnoses, intervention targets, and effective drugs to treat IBD.

Oxidative stress (OS), redox signaling, inflammation, and electrophilic stress are signaling pathways closely associated with the development of the IBD disease process [7]. Among them, OS is considered as one of the main pathophysiological mechanisms of IBD [8]. As we all know, the production and release of reactive oxygen species (ROS) are directly related to chronic active inflammation. The sustained release of ROS in the local microenvironment of actively inflamed mucosal lesions perpetuates intestinal inflammation and leads to increased tissue destruction [9]. OS is a state of imbalance between ROS production and elimination, which can occur in the inflamed intestinal mucosa and even extend into the deeper layers of the intestinal wall [10]. The excessive production of ROS in IBD can be quantified by measuring specific components of the whole-body redox state [11]. Nuclear factor erythroid 2 (NF-E2)-related factor 2 (Nrf2), a known factor that regulates ROS, is considered a promising therapeutic target for the treatment of various gastrointestinal disorders, and can prevent intestinal diseases by regulating antioxidant enzymes and proinflammatory cytokines to maintain redox homeostasis. Once Nrf2 dissociates from Kelch-like ECH-related protein 1 (Keap1) in the cytoplasm, Nrf2 translocates to the nucleus, binds to antioxidant response elements, and then increases the expression of antioxidant enzyme (e.g., HO-1, NQO1, and SOD) expression and inhibits the growth of cellular pro-inflammatory factors TNF-α, IL-6, IL-1β, and INOS [12]. To date, a large number of studies have demonstrated the involvement of Nrf2 in the regulation of IBD by enabling redox regulation and inhibiting inflammation and tissue damage [13,14,15,16]. Heme oxygenase-1 (HO-1) of the heme oxygenase family is a gene regulated by Nrf2. As one of the phase II detoxification enzymes downstream of Nrf2, elevated cellular levels of HO-1 are thought to be an antioxidant mechanism, in order to protect cells from ROS attacks [17]. Therefore, the Nrf2/HO-1 axis has emerged as an important target for the antioxidant stress response in IBD. In this review, we aim to elucidate the key role of the Nrf2/HO-1 signaling pathway in IBD and IBD complications, as well as the potential of phytochemicals to treat IBD through this pathway.

## 2. Antioxidant Nrf2/HO-1 Signaling Pathway

### 2.1. Structure and Function of Nrf2

**Figure 1:**
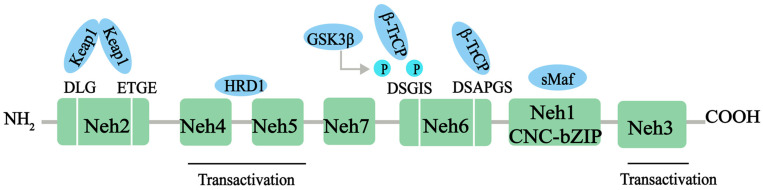
The protein structure of Nrf2. The Nrf2 protein contains 7 domains, Neh1-7. The N-terminal Nrf2-ECH homology (Neh) 2 domain has ETGE and DLG motifs that bind to the Kelch domain of Kelch-like ECH-associated protein 1 (KEAP1). The Neh4 and Neh5 domains mediate the transactivation activity of Nrf2. The Neh7 domain inhibits the transcription of the Nrf2 target gene. The Neh6 domain has DSGIS and DSAPGS motifs. The sMaf element of the Neh1 domain helps Nrf2 dimerization with DNA binding to other transcription factors. The C-terminal Neh3 domain supports Nrf2 transcriptional activity. NH2, N-terminal; COOH, C-terminal; GSK3-β, glycogen synthase kinase-3 beta; β-TrCP, beta-transducin repeat containing E3 ubiquitin protein ligase; HRD1, E3 ubiquitin ligase; sMaf, small musculoaponeurotic fibrosarcoma protein.

Nrf2, a member of the Cap‘n’collar (CNC)-BZIP family of transcription factors, is a major transcriptional regulator of antioxidant response, controlling the expression of a range of antioxidant proteins, such as heme oxygenase-1 (HO-1), superoxide dismutase (SOD), and glutathione (GSH), to reduce the level of ROS and exert antioxidant effects. Nrf2 contains 605 amino acids and is divided into seven effective Nrf2-ECH homology (Neh) domains (Neh1-7), each with different functions (Figure 1). The Neh1 structural domain contains the bZIP motif, which enables Nrf2 to bind to the antioxidation response element (ARE) sequence. In addition, Neh1 can interact with UbcM2 (E2 ubiquitin-conjugating enzyme) to regulate the stability of the Nrf2 protein. N-terminal Neh2 is a redox-sensitive domain, consisting of seven lysine residues responsible for ubiquitination. The ETGE and DLG motifs of Neh2 are involved in Keap1 binding, which leads to Cullin 3-dependent E3 ubiquitination and proteasomal degradation. The C-terminal Neh3 domain is necessary for Nrf2 transcription activity, and helps Nrf2 to be transactivated through the interaction with the chromogenic ATPase/deconjugate DNA-binding protein (CHD6) [18,19]. Neh4 and Neh5 domains are rich in acid residues and have been proved to interact with the CH3 domain of the cAMP response element binding protein (CREB) binding protein (CBP), which is essential for the Nrf2-mediated transactivation of ARE [20]. The redox-insensitive Neh6 domain is characterized by the ubiquity of serine residues with DSGIS and DSAPGS motifs. The Neh6 structural domain provides a phosphorylation-dependent binding platform for β-TrCP and is responsible for the regulation of Nrf2 degradation through a mechanism independent of KEAP1 [21]. Finally, Neh7 can inhibit the expression of the Nrf2 target gene by interacting with Nrf2 inhibitor retinal X receptor α [22].

Under normal conditions, Nrf2 can combine with Kelch-like ECH-related protein 1 (Keap1) to form the Keap1/Nrf2 complex. These complexes are anchored in the cytoplasm by actin and are in a low-activity state [23]. Under oxidative stress or other pathological stimuli, the cysteine residue of Keap1 is modified to induce Nrf2 phosphorylation, and Nrf2 is released from the complex and translocated to the nucleus [24]. The sequence of AREs in the nucleus can accurately recognize NRF4-MAF and bind to the Neh2 domain. The antioxidant response element (ARE) sequence in the nucleus accurately recognizes NRF4-MAF and binds to the Neh2 structural domain. Then, with the help of the cAMP response element binding protein (CREB) and transcriptional activator, it initiates Nrf2-mediated transcription; activates a series of antioxidant enzymes and phase II antioxidant enzymes, such as HO-1, NAD(P)H: quinone oxidoreductase 1 (NQO1), superoxide dismutase (SOD), and glutathione peroxidase (GSH-Px); and removes harmful substances, such as ROS, to exert antioxidative, anti-inflammatory, and anti-apoptotic cytoprotective effects [25].

### 2.2. Structure and Function of HO-1

Heme oxygenase (HO) is an enzyme encoded by the HMOX gene, which was described by Tenhunen and Schmid in 1968, and can catalyze the degradation of heme to carbon monoxide (CO), iron, and bilirubin [26]. HO consists of three forms, HO-1 or heat shock protein 32 (Hsp32) is an inducible form, HO-2 has constitutive expression, and HO-3 appears to be a pseudogene derived from HO-2 transcripts expressed only in rat neurons [27]. Of these, HO-1, called HMOX1, is located on chromosome 22q12.3. It has five exons, four introns, and three regulatory regions, one proximal (−0.3 Kb) and two distal promoter regions (−4 Kb for E1 and −10 Kb for E2) [28]. These regulatory regions contain different transcription factor binding sites (hypoxia-inducible factor 1, nuclear factor κB (NF-κB), activator protein binding site, stress response element (StRE), metal response element, and heat shock consensus sequences), which are involved in the regulation of cellular redox status [29], as a result of or downstream of Nrf2 activation, if exposed to cellular stress inducers, such as oxidants, inflammatory chemokines/cytokines, and tissue injury [30]. Studies have shown that an increased expression of HO-1 contributes to lower levels of inflammation-associated cytokines, such as TNF-α, IL-6, and IL-1β, which in turn attenuates the inflammatory response [31,32].

HO-1 is an inducible homolog influenced by heme products. The enhancement of HO-1 activity reduces the amount of free heme, thereby preventing the production of ROS and reducing cellular damage. It can be seen that the degradation of heme by HO-1 is helpful to maintain intracellular homeostasis and represents the direct cytoprotection of HO-1 [33]. However, HO-1 over-activation may lead to iron-dependent death, termed ferroptosis [34]. Ferroptosis is a novel form of regulatory cell death that results from iron accumulation leading to lipid peroxidation and glutathione depletion [35]. Low or moderate HO-1 activity removes the heme group (a potent oxidant) and neutralizes ROS, allowing the produced iron to enter a non-promotional oxidative state, providing cytoprotection. However, when HO-1 is highly activated, NADPH levels are reduced and iron levels are elevated, and ferritin neutralization is overwhelming, compared to unstable iron, leading to an increase in the pro-oxidative free iron pool [36]. Unable to offset the iron oxide pool will increase the amount of ROS and HO-1 activities and the consumption of antioxidants (such as glutathione), which will eventually lead to ferroptosis [37]. Several studies on HO-1-deficient mouse models and human HO-1 deficiency caused by the HMOX1 mutation have emphasized the importance of HO-1 for tissue iron and organ homeostasis, antioxidant stress, and macrophage function [38].

### 2.3. Antioxidant Effect of the Nrf2/HO-1 Signal

**Figure 2:**
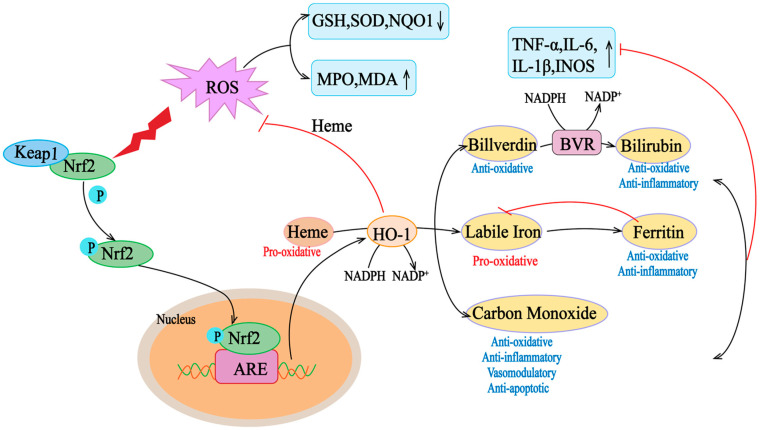
Nrf2/HO-1 axis activity in oxidative conditions. Heme is a pro-oxidant and generates reactive oxygen species (ROS). Under oxidative stress, Keap1 undergoes a conformational change that induces its ubiquitination and degradation, as well as the translocation of Nrf2 to the nucleus Nrf2, which translocates to the nucleus and binds to the ARE in the promoter region of antioxidant genes. Elevation of the target gene downstream of Nrf2, HO-1, helps to scavenge mROS, thus counteracting the deleterious effects of ROS on mitochondrial function. HO-1 degrades heme to bilirubin and carbon (CO). HO-1 degrades hemoglobin to biliverdin, unstable iron, and carbon monoxide (CO). Unstable iron can act as a pro-oxidant via the Fenton reaction, but it stimulates ferritin transcription, which stores it for antioxidant and anti-inflammatory activity. Carbon monoxide is involved in several downstream processes, ultimately acting as an antioxidant, anti-inflammatory, vasomodulator, and anti-apoptotic agent. Biliverdin is an antioxidant molecule that is rapidly converted to bilirubin by biliverdin reductase (BVR), which has antioxidant and anti-inflammatory properties. ARE, antioxidant response element; ROS, reactive oxygen species; GSH, glutathione; INOS, inductible nitric oxide synthase; IL, interleukin; MPO, myeloperoxidase; MDA, malondialdehyde; NO, nitric oxide; NQO1, NAD(P)H: quinone oxidoreductase; ROS, reactive oxygen species; SOD, superoxide dismutase; TNF-α, tumor necrosis factor α. The “↑” and “↓” in the picture indicate increase and decrease respectively.

Multiple regulatory elements allow HMOX1 transcription in response to excessive oxidative and inflammatory stimuli, such as heme, ROS, growth factors, and cytokines, such as IL-6, IL-1β, TNF-α, and INOS [39]. Although the regulatory elements of HO-1 are various, the main sequence motif is StRE, which is similar to the Maf reaction element and antioxidant reaction element (ARE) [38]. Oxidative stress leads to the release of heme from the hemoprotein pocket, which is transcribed for HMOX1 through multiple signaling pathways and transcription factors. Free heme binds to BTB and CNC homolog 1 (Bach1), a transcriptional repressor of HMOX1, and induces a conformational change in its structure, leading to the unbinding of Bach1 from the StRE element and an increase in the transcription of HO-1 in cells [40]. At the same time, elevated ROS levels lead to the separation of Keap1 and Nrf2, which enables Nrf2 to translocate to the nucleus and bind to the antioxidant response element (ARE) of the HOMX1 gene. With the release of Bach1 from the StRE of the gene, Nrf2 binds to ARE, and HMOX1 transcription increases [41]. Thus, Nrf2 and Bach1 activate or repress HMOX1 transcription, respectively, and play key roles in HMOX1 regulation (Figure 2). Therefore, HO-1 is a gene regulated by Nrf2, and Nrf2/HO-1 is one of the most critical cell protection mechanisms that are activated to maintain antioxidant/oxidative homeostasis during cellular stress [42].

Nrf2/HO-1 can play different roles in the crosstalk with other pathways. The increase in ROS levels caused by various reasons can enhance inflammatory pathways by upregulating pro-inflammatory cytokines via NF-κB and enhancing their activation and translocation [43]. Meanwhile, pro-inflammatory molecules also cause NF-κB activation by stimulating the IkappaB kinase/NF-κB complex. In addition, ROS increases interleukin-1β (IL-1β) and IL-18 levels by inducing NLR-containing pyrin structural domain 3 (NLRP3) [44]. However, such pro-oxidant stimulation activates the Nrf2/HO-1 signaling pathway such that Nrf2 stimulates HMOX-1 transcription and enhances levels of HO-1, which degrades heme-generated CO and bilirubin to inhibit apoptosis by suppressing mitochondrial dysfunction and reducing H_2_O_2_ levels [45].

## 3. Role of Nrf2/HO-1 in Normal Gut Development and Normal Gut Function

### 3.1. Role of Nrf2/HO-1 in Normal Intestinal Development

During the embryonic development of mice, the primitive gastrointestinal tract (tube) is established around day 9.5 of embryonic development. After its growth, starting at day 14.5 of embryonic development, it is the key link to form a normal hindgut, including epithelial remodeling, transient emergence of villi, and the formation of crypts and stem cell niches [46]. The coordination between Notch and Wnt signal pathways drives intestinal development, and Nrf2 can affect the activation of these signal transduction pathways [47,48]. It has been demonstrated that the crosstalk between Nrf2 and Notch pathways regulates gastrointestinal maturation. The downstream effector of Notch, the Math1 gene promoter, has a functional ARE sequence in its proximal region, so the negative transcriptional regulation of Math1 activates Nrf2 signaling in the intestinal epithelium, leading to intestinal elongation and extension [49]. Meanwhile, Notch-1 and its related genes are attenuated in the embryonic fibroblasts of Nrf2 knockout mice [50]. Furthermore, Wnt controls the differentiation of transit and expansion cells to Paneth cells in the small intestine and contributes to the maintenance of the mucosal barrier, yet Wnt is activated during oxidative stress associated with Nrf2 deficiency [51]. A study confirmed that β-collagen in the Wnt pathway can activate the Nrf2 pathway, while Nrf2 can strongly inhibit β-collagen. In addition, β-TrCP1 binds to β-connexin, thereby attenuating the inhibitory effect of Nrf2 on β-connexin [52]. Chen et al. found high levels of Nrf2 mRNA on the luminal side of the intestinal tract during pregnancy in mice and a significant difference in the levels of Nrf2 mRNA between the organ and gestation day [53]. Recent studies have also demonstrated a sustained increase in Nrf2 levels in the intestine but a decrease in other tissues (e.g., lungs or heart) after 14.5 to 18.5 days of embryonic development [46]. Nrf2 transcriptional deletion leads to the significant elongation of the colon, altered distribution of crypts, enlarged cups, and a significant increase in mucin levels [54]. Thus, Nrf2 transcriptional activity is an indispensable regulatory mechanism in intestinal development, regulating enterocyte proliferation and differentiation at different stages of the embryo [55].

HO-1 deficiency leads to embryo death, and some studies have evaluated the role of HO-1 in embryo survival. HO-1 is already expressed in the placenta and pregnant uterus at the 6th day of embryo (E6.5). The placenta of the E14.5 embryo expresses very high levels of HO-1 mRNA, protein, and activity [56]. The intrauterine mortality of HO-1 knockout animals is very high, and there is serious chronic inflammation in general, accompanied by oxidative stress and susceptibility to iron metabolism dysfunction [57,58]. Smooth muscle cells (SMCs) belong to the mesoderm lineage during the development of embryonic stem cells [59]. SMα-actinin is the main component of contractile devices in many organ systems (including vascular, respiratory, and gastrointestinal systems), which contributes to tissue structure and function [60]. It has been shown that, during the differentiation of SMCs into SMα-actinin, the lack of HO-1 significantly increased the level of ROS at day 3, while leading to a decrease in CO concentration and a further increase in ROS [61]. HO-1 acts through reaction products, such as CO and bilirubin, and HO-1 deficiency accelerates and enhances the expression of SMα-actinin in the mesoderm [61]. Generally speaking, HO-1 plays a key role in regulating ROS levels and germ layer differentiation (especially the endoderm and mesoderm) during early differentiation [62]. Hypoxia-induced delayed expression of HO-1 can easily lead to intestinal injury, and low HO-1 is closely associated with intestinal barrier failure in animal models of intestinal injury [63].

### 3.2. Role of Nrf2/HO-1 in Normal Intestinal Function

The intestinal epithelial tight junction (TJ) is the most important structure of the mechanical barrier, which is responsible for regulating paracellular channels, modulating intestinal mucosal permeability, and preventing the entry of antigenic substances into intestinal mucosa [64]. It is mainly composed of transmembrane proteins, such as occludin, claudin, and junction adhesion molecules (JAMs). The TJ together with junction complex proteins (ZO-1, ZO-2, ZO-3, etc.) and the cytoskeleton (microtubules, microfilaments, and filaments) form a tight junction complex [65]. The integrity of the TJ determines the integrity and permeability of the intestinal epithelial barrier [66]. An abnormal expression of the TJ can directly lead to intestinal barrier dysfunction, which is manifested by the increase in intestinal mucosal permeability, and pathogenic microorganisms enter the lamina propria of mucosa through epithelial cells, causing sustained immune and inflammatory reactions in the intestinal mucosa [67].

The expression of the TJ protein is closely related to the Nrf2/HO-1 pathway [68]. It was shown that Nrf2 could enhance the TJ in the intestinal epithelium, and even activated with ERK/Nrf2/HO-1 signaling cascade-enhanced occludin and ZO-1 proteins in the intestinal epithelium [69]. At the same time, the Nrf2/HO-1 signaling pathway can inhibit NF-κB signaling and restore the LPS-induced expression of occludin, ZO-1, and claudin-5 in Caco-2 cells [70]. The prevailing view is that Nrf2/HO-1 is involved in the mechanism of the TJ′s protection by promoting the regulation of intestinal epithelial cell autophagy mainly by preventing apoptosis and ferroptosis [71,72]. Nrf2 deficiency leads to mitochondrial dysfunction, down-regulates claudin-4 expression, and impairs esophageal epithelial tight junctions. But, the activation of Nrf2 inhibits apoptosis in intestinal epithelial cells and stimulates autophagy to enhance the protection of tight junction proteins [73]. Another study confirmed that ERK/Nrf2/HO-1-mediated stimulation mitochondrial autophagy can improve intestinal mucosal injury and barrier dysfunction [69]. In addition, recent studies show that activating the Nrf2/HO-1 pathway can also inhibit OS to significantly alleviate ferroptosis in intestinal epithelial cells, upregulate TJ proteins, and enhance the integrity of the intestinal barrier in a UC mouse model [74,75]. Therefore, Nrf2/HO-1, as a key coordinator to maintain the homeostasis of the intestinal intracellular environment, plays an important role in maintaining the balance of coordinated oxidative stress and protecting the intestinal mucosal barrier in normal intestinal midgut epithelial cells in a normal intestine.

## 4. Role of Nrf2/HO-1 in IBD and IBD Complications

### 4.1. Role of Nrf2/HO-1 in IBD

At present, the mainstream view is that the main mechanism of the Nrf2/HO-1 signaling pathway in the treatment of IBD involves controlling oxidative stress and reducing inflammatory damage [76,77], regulating intestinal microbiota to repair the intestinal mucosal barrier [78,79], and preventing the ferroptosis of intestinal epithelial cells [80,81].

#### 4.1.1. Nrf2/HO-1 Attenuates Intestinal Inflammation and Injury by Controlling Oxidative Stress

Over the years, relevant studies on the Nrf2/HO-1 axis participating in IBD have shown that the activation of Nrf2 is a key event in normal cell homeostasis. The expression level of HO-1 in healthy intestines is low, but it will be significantly induced in triggering inflammatory states [82,83,84,85]. As early as 2001, Wang et al. believed that HO-1 was an important antioxidant and anti-inflammatory enzyme regulated by Nrf2, which played a protective role in trinitrobenzene sulfonic acid-induced colitis in rats [86]. Evidence for the involvement of the Nrf2/HO-1 axis in the course of IBD was first published in 2006. Khor and colleagues demonstrated that DSS-induced colitis is associated with an increased expression of Nrf2 regulatory enzymes (e.g., HO-1, NQO1, UGT1A1, and GSTM1), so the increased susceptibility of Nrf2-deficient mice to DSS-induced colitis may be due to the decreased expression of antioxidant and phase II detoxification enzymes, such as HO-1, along with the increased expression of proinflammatory mediators [87]. The levels of pro-inflammatory cytokines and lipid peroxidation in the colon of Nrf2 knockout mice increased significantly, while the expression level of antioxidant enzymes decreased [88]. These studies indicate that the regulation of the Nrf2/HO-1 signaling pathway may represent a promising avenue to treat IBD.

**Figure 3:**
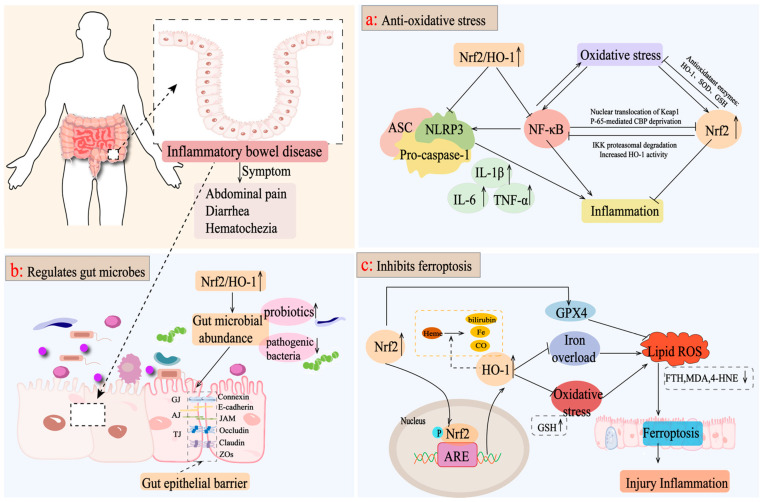
Mechanisms of targeting the Nrf2/HO-1 axis for the treatment of IBD. (**a**) Nrf2/HO-1 activates a series of signaling pathways targeting inflammation, such as the NF-κB pathway and the NLRP3 pathway, to reduce the levels of inflammatory factors, such as IL-6, TNF-α, and IL-1β, as well as inducing the production of antioxidant enzymes, such as SOD and GSH, to reduce the oxidative stress caused by the aggravation of ROS and to attenuate the pathologic inflammatory response of the intestine. (**b**) Activation of the Nrf2/HO-1 axis improves the intestinal microbiota, promotes the growth of beneficial bacteria, inhibits the colonization of pathogenic bacteria, increases the expression level of tight junction proteins, such as intestinal occludin, claudin-1, and ZO-1, and maintains the integrity of the intestinal epithelial barrier. (**c**) Activation of Nrf2/HO-1 upregulates GPX4 expression, inhibits oxidative stress and iron overload, and reduces ROS production, thereby inhibiting intracellular iron death and exerting anti-inflammatory effects.

In IBD, the Nrf2/HO-1 pathway can reduce intestinal inflammation and injury and protect intestinal integrity by controlling oxidative stress [89]. Nrf2/HO-1 reduces the aggravation of ROS by regulating inflammatory mediators and inducing antioxidant enzyme production, such as SOD, and GSH to induce oxidative stress damage and attenuating pathological inflammatory responses [90]. Moreover, the activation of Nrf2/HO-1 can activate a series of signal pathways targeting inflammation, such as the NF-κB pathway and NLRP3 pathway, to balance cell homeostasis. According to the research, by regulating the interaction between Nrf2/HO-1 and NF-κB, oxidative stress, inflammatory reactions, and apoptosis in acetic acid-induced UC rats would be suppressed [90,91]. In addition, activating the COX-2/Nrf2/HO-1 pathway inhibits NF-κB phosphorylation and NLRP3 inflammatory vesicle activation, activates the expression of Pink1/Parkin to promote the occurrence of mitochondrial autophagy, and reduces the secretion of corresponding inflammatory factors to improve intestinal inflammation [92,93]. In conclusion, the activation of Nrf2/HO-1 in the intestine can inhibit the inflammatory pathway or reduce the overreaction of oxidative stress, thus reducing intestinal injury and inflammation (Figure 3a).

#### 4.1.2. Nrf2/HO-1 Promotes the Maintenance of the Intestinal Epithelial Barrier by Regulating Intestinal Microbiota

Intestinal microbiota imbalance includes changes in intestinal microbiota composition and metabolism, which will promote the development of chronic diarrhea, inflammatory bowel disease, colon cancer, and other diseases, and is closely related to the occurrence and progress of IBD [94]. IBD is the result of the dysregulation of immune response and oxidative homeostasis, which may lead to an imbalance of gut microbial ecology [95]. It is reported that *Lactobacillus casei* ATCC 393 combined with vasoactive intestinal peptide increased the abundance of *Firmicutes* and *Turicibacter*, decreased the abundance of *Bacteroides*, significantly elevated the expression levels of occludin and claudin-1, and effectively alleviated DSS-induced intestinal barrier dysfunction through the NF-κB and Nrf2 signaling pathways [96]. *Rice protein* and its rice protein peptide activated the Keap1/Nrf2 signaling pathway, mediated the downstream expression of HO-1, increased the relative abundance of beneficial bacteria, such as *Akkermansia*, and adjusted the level of short-chain fatty acids (such as propionic acid, acetic acid, *n*-butyric acid, *n*-valeric acid, *i*-butyric acid, and *4*-methylvaleric acid) to increase the level of intestinal tight junction proteins and maintain intestinal mucosal barrier function [97,98]. Therefore, the increase in nuclear Nrf2 and HO-1 protein levels is considered as a response index for the effective treatment of IBD. For example, *Bruguiera gymnorrhiza fruit* promoted the growth of probiotic bacteria (*Bifidobacterium, Anaerotruncus, and Lactobacillus*) in the intestine and inhibited the colonization of pathogenic bacteria (*Bacteroides* and *Streptococcus*) by upregulating the protein levels of nuclear Nrf2 and HO-1, and inhibiting the protein expression of Keap1 and cytoplasmic Nrf2, which contributed to maintaining IBD mouse intestinal homeostasis [99]. Similarly, *Tetrastigma hemsley anum leaves* improved the expression of SOD, CAT, HO-1, NQO1, and GCLC by activating the nuclear transfer of Nrf2, and adjusted the imbalance of intestinal microbiota structure by increasing the expression level of tight junction proteins, such as intestinal occlusive protein, claudin-1, and ZO-1 [100]. Therefore, the activation of the Nrf2/HO-1 axis is helpful to maintain intestinal microbial ecological homeostasis and preserve the integrity of the intestinal epithelial barrier, while further studies are needed to elucidate the underlying mechanisms involved (Figure 3b).

#### 4.1.3. Nrf2/HO-1 Prevents Ferroptosis in Enterocytes

Intestinal cells can rely on antioxidant enzymes and molecules to resist a certain level of ROS under physiological conditions, but when the antioxidant defense system fails to counteract the overproduction of ROS, the cells will experience lipid peroxidation, protein dysfunction, and eventually death [101]. Therefore, inhibiting intestinal cell death by regulating OS may be an effective intervention to alleviate IBD. Ferroptosis is a recently recognized form of regulatory cell death, which is caused by the iron-dependent accumulation of lipid reactive oxygen species [102]. Ferroptosis is mainly driven by the inactivation of the antioxidant system, and closely related to OS [103]. Nrf2 exhibits anti-inflammatory, antioxidant, and anti-ferroptosis properties, and targeting the Nrf2/HO-1 pathway is an effective approach to attenuate ferroptosis in IBD [104].

The activation of the Nrf2/HO-1 signaling pathway inhibits intracellular ferroptosis, which is characterized by decreased ROS production and the down-regulation of iron overload [105,106]. The abnormal expression of FTH1 eventually leads to the collapse of cell antioxidant defense, resulting in iron storage dysfunction and cell death [107]. Chen et al. proved that the NRF2/HO-1 pathway mediated by *Astragalus polysaccharide* inhibited the decrease in ferroptosis-related genes (PTGS2, FTH, and FTL) in DSS-induced mice and prevented ferroptosis in a human Caco-2 cell model [108]. GPX4 is a major target of ferroptosis, which promotes the reduction of lipid peroxide in cells under ferroptosis conditions [109]. Knockdown of GPX4 can induce ROS accumulation and subsequent ferroptosis [110]. *Gingerenone A* upregulated GPX4 expression in DSS-induced mice by increasing Nrf2 and HO-1 proteins, and significantly attenuates ferroptosis secondary to hepatic injury in DSS-induced colitis mice [111]. GSH is the most abundant antioxidant in the cell, and its content is negatively correlated with ferroptosis, which can be catalyzed by GPX4 to scavenge intracellular ROS [112]. *Dandelion polysaccharide* (DP) increased the expression levels of Nrf2, HO-1, NQO-1, and GSH, and reduced MDA and 4-HNE markers in UC mice. It suggested that activating Nrf2/HO-1 by DP is beneficial to inhibit the ferroptosis process of intestinal epithelial cells in UC mice, enhances the integrity of the intestinal barrier, and plays a key role in enhancing antioxidant potential [74]. However, Study believes that the over-activation of Nrf2/HO-1 would disturb the balance of iron ion metabolism to induce ferroptosis. Chen et al. showed that, after the administration of ferritin-1, a small molecule inhibitor of ferroptosis, reversed the DSS-induced high expression of Nrf2 and HO-1 in a DSS-induced animal model of IBD. In addition, the expression of COX2 and ACSL4 decreased obviously, and the expression levels of GPX4 and FTH1 improved, which was considered to be related to the improvement of UC by inhibiting ferroptosis through the Nrf2/HO-1 pathway [80]. To sum up, targeting the Nrf2/HO-1 pathway in the intestine is closely related to ferroptosis in IBD, especially in UC, but there is little research on Crohn’s disease, and more experimental basic research is needed to support it (Figure 3c).

### 4.2. Role of Nrf2/HO-1 in the Complications of IBD (Intestinal Fibrosis and CRC)

#### 4.2.1. Role of the Nrf2/HO-1 Pathway in Intestinal Fibrosis in IBD 

Intestinal fibrosis develops from chronic, recurrent, or unresolved intestinal inflammation causing persistent tissue damage and the failure to rebuild tissue structure [113]. Intestinal fibrosis is a common and serious complication of IBD, as evidenced by the excessive deposition of extracellular matrix and loss of normal function [114]. Intestinal fibrosis leads to increased wall stiffness, stenosis formation, and obstruction, which is one of the biggest problems in IBD [115]. However, there may be differences in intestinal fibrosis between CD and UC patients. During UC attack, the accumulation of the extracellular matrix (ECM) in mucosa and submucosa leads to the thickening of muscular mucosa, accompanied by the shortening of the colon and increased stiffness [116,117]. In addition, the fibrosis of UC is related to inflammation and epithelial destruction, such as the injury of TJs [118]. On the contrary, CD is caused by excessive ECM deposition due to abnormal inflammatory stimulation and uncontrolled activation of mesenchymal cells [119].

The abnormal deposition of the ECM caused by the unbalanced production and degradation of the ECM is characteristic of fibrosis. The degradation of the ECM is mainly carried out by matrix metalloproteinases (MMPs), which are counteracted by tissue inhibitors of MMPs. Therefore, the imbalance between MMPs and their inhibitors seems to be related to the increase in ECM deposition and subsequent tissue fibrosis [120]. In IBD patients, MMP activity is increased and is usually lower in CD patients compared with UC patients [121]. Since ECM degradation is regulated by MMPs and TIMPs, the imbalance of these enzyme activities may lead to ECM deposition and fibrosis [122]. More and more evidence has clarified the role of MMP-3 in IBD, especially the concentration of MMP3 that is critical for determining responsiveness to infliximab therapy in individuals diagnosed with IBD [123,124]. Therefore, reducing the specific inhibition of MMPs by modulating the Nrf2/HO-1 signaling pathway may have positive significance for the treatment of IBD-induced intestinal fibrosis.

It is well known that the TGFB--SMAD pathway serves as a target for antifibrotic therapy to promote extracellular matrix protein deposition and fibroblast transformation [125]. TGF-β transcription is regulated by Nrf2. Previous studies have shown that Nrf2 could inhibit the TGF-1β/SMAD pathway to alleviate fibrosis by scavenging ROS and reducing the accumulation of oxidative stress substances [126]. The administration of Nrf2 agonists suppressed TGF-β levels in a mouse model of 2,4,6-trinitrobenzene sulfonic acid (TNBS)-induced chronic colitis [127]. In addition, Nrf2 has been proved to prevent the transformation of intestinal fibroblasts to myofibroblasts in vivo and in vitro by inhibiting the ROS-dependent TGF-β1/SMADs pathway [128,129]. For example, Nrf2 protein and mRNA levels were upregulated in CCD-18Co, a normal human colonic fibroblast, which negatively regulated the TGF-β1/SMADs pathway to inhibit intestinal fibrosis [130,131]. These findings emphasize the potential of Nrf2 activation in limiting the TGF-β1/Smad signal transduction axis and subsequently alleviating intestinal fibrosis.

#### 4.2.2. Role of the Nrf2/HO-1 Pathway in IBD-Associated Colorectal Cancer

Colorectal cancer (CRC) is the third most common cancer in the world. As developing countries continue to progress, the incidence of colorectal cancer associated with IBD is about 2%, and the global incidence of colorectal cancer is expected to increase to 2.5 million new cases by 2035 [132]. Pathogenetic features, such as chromosomal and microsatellite instability, and DNA hypermethylation have been reported in patients with sporadic CRC (sCRC) and in patients with IBD-associated CRC (IBD-CRC) [133]. Both types of cancer share common functional driver genes, such as APC, P53, MYC, KRAS, PIK3CA, SMAD4, and ARID1A. Sporadic colorectal cancers originate from adenomatous (and sessile serrated polyps) precursors that progress through various stages to carcinomas in a classic “adenoma carcinoma sequence”. A specific subtype of CRC, the so-called colitis-associated colorectal cancer (CAC), may also originate from chronic inflammation in the course of IBD. The lifetime risk of CAC occurrence has been estimated to be increased by up to 20% in patients with IBD [134]. Although the incidence of IBD-CRC is decreasing, its prognosis is still worse than that of sporadic CRC [135]. IBD-associated CRC follows different genetic and molecular pathogenic pathways than sporadic CRC and can be considered a complication of chronic intestinal inflammation. IBD-CRC is considered a unique disease compared to sCRC, and little is known about the pathogenesis of IBD-CRC [136]. Chronic inflammation leading to heteroplasia is a key driver of tumor alteration and progression, and is also considered the most critical risk factor for the development of colitis-associated CRC. Chronic inflammation produces oxidative stress-induced damage to DNA, which activates tumor-promoting genes and inactivates tumor suppressor genes [137]. Thus, unlike sporadic CRC, IBD-CRC does not follow the traditional adenoma–carcinoma sequence; instead, it can progress from low-grade to high-grade dysplasia and ultimately to CRC, i.e., the “inflammation dysplasia carcinoma sequence” [138].

NRF2 has been shown to act as both a tumor suppressor and an oncogene. Even though the protective effect of Nrf2 against carcinogenesis has been well demonstrated in normal cells, Nrf2 transcription induced by oncogenes (K-Ras, B-Raf, or Myc) promotes cancer growth by exerting a protective effect on tumor cells [139]. Meanwhile, this pro-cancer behavior can be explained by mutations in NRF2 or its upstream controller KEAP1, which leads to hyperactivity, or by epigenetic modifications of Nrf2 [140]. In sporadic CRC, a study evaluated the expression of Nrf2 at the RNA and protein levels, and Gfer at the mRNA level, in the intestinal mucosa of patients with colorectal adenomas (the SpCA group) or without proliferative lesions (the control group), in order to determine whether there was an association between the expression of these two genes and the development of colorectal adenomas. The results clearly showed that the Nrf2 gene and protein expression were significantly lower in the control group compared to the control group, which was associated with the significantly lower expression of the Gfer gene in the SpCA group, a result that demonstrates that mechanisms associated with the reduced antioxidant and anti-inflammatory activity of the Nrf2 gene may play a causative role in the development of pre-cancerous lesions as a cumulative risk for a predisposition to the progression to CRC [141]. In addition, a study tested the effect of the 2-HOBA modulation of NRF2 on colon carcinogenesis in CAC and sCRC models. The results showed fewer and smaller adenomas in the 2-HOBA-treated group, while moderate inflammation in non-tumor areas was unaffected. The NRF2 target gene Slc7a11 was down-regulated in tumors of mice administered 2-HOBA compared to sham treatment. A strong nuclear translocation of NRF2 was observed in the CEC of mice treated with tamoxifen compared with the controls; however, in 2-HOBA-treated mice, there was less nuclear translocation. Therefore, the nuclear translocation of NRF2 and the expression of NRF2 target genes in CEC are affected by Apc deletion and 2-HOBA treatment, suggesting that 2-HOBA may be involved in repressing the transcriptional mechanism of NRF2 to alleviate colon carcinogenesis [142].

In IBD-CRC, various data show that the long-term existence of inflammatory bowel disease, especially chronic ulcerative colitis, can lead to a malignant transformation to colon cancer and even promote the progression and early metastasis of colon cancer [136]. The activation of the Nrf2/HO-1 axis is a double-edged sword regarding cancer [143]. The Nrf2/HO-1 axis plays a protective role in the early stage of CRC development [144], but it is also associated with the processes of cancer cell proliferation, metastasis, and radiotherapy resistance in the advanced tumor stage [145,146,147].

The activation of the Nrf2/HO-1 axis plays a protective role in the early stage of CRC development, protecting cells from intracellular oxidative stress and inflammatory reaction. Ensuring the proper basal regulation of the Nrf2/HO-1 axis is essential to avoid carcinogenesis in colon tissues [148]. Existing research shows that miR-222-3p is the main regulator of oxidative stress. By activating Nrf2/HO-1 signal transduction and inhibiting miR-222-3p in intestinal epithelial cells, oxidative stress could be reduced, and colitis-related colorectal cancer could be improved [149]. Melatonin reduces autophagy and delays progression in the colon of mice with colitis-associated colon carcinogenesis (CACC) by ameliorating inflammation and Nrf2/HO-1-mediated oxidative stress in the colon of CACC mice [150]. This suggests that identifying the molecules that keep the Nrf2/HO-1 axis constant may be helpful to prevent the development of cancer in the early stage [151].

The activation of the Nrf2/HO-1 axis is closely related to the proliferation and metastasis of anticancer cells in the advanced tumor stage [152]. As we all know, the Nrf2 pathway is regarded as a double-edged sword. HO-1, as a key enzyme to remove toxic heme downstream of the Nrf2 pathway, is one of the clear state indicators of the duality of this pathway [22]. VEGF is an angiogenic factor released by malignant colon cells, which can stimulate the growth of blood vessels in tumors, leading to the rapid growth and spread of malignant cells, and shows a high level of activity in the early and advanced (metastatic) stages of colon cancer [153]. It was found that VEGF expression was elevated in the colon tissues of AOM-induced colon cancer in rats and was positively correlated with a significant increase in new blood vessels and ACF formation [154]. The inhibition of the Nrf2 pathway could block the accumulation of HIF-1α in colon cancer cells, inhibit the expression of VEGF and HIF-1α target genes, and reduce the growth of xenograft tumors and angiogenesis under hypoxic conditions in mice [155]. M2 tumor-associated macrophages (M2-TAMs) are one of the key immunosuppressive cells that influence cancer cell proliferation and metastasis. A clinical study showed that, compared with M1- TAMs and M1/M2-TAMs, the expression levels of Nrf2 and HO-1 in the M2-TAMs of the tumor margin were significantly increased, and the number of Nrf2 or HO-1 M2-TAMs in the tumor matrix was significantly increased. Thus, the increased frequency of M2-TAM infiltration in the colorectal cancer tumor microenvironment is associated with antioxidative stress mediated by the Nrf2-HO-1 axis [156]. The heterocyclic organobismuth (III) compound is a bismuth compound that can effectively resist the proliferation activity of various cancer cell lines. The first study to report the induction of the NRF2-HO-1 axis by an organobismuth (III) compound showed that compound III activated the NRF2/HO-1 pathway in the human colon cancer cell line DLD-1, increasing HO-1 expression and leading to the death of colorectal cancer cells [157]. Meanwhile, Fang et al. found that Quyujiedutang (QYJD), and *Oxymatrine* and *Matrine* in it, increased the protein levels of Nrf2 and HO-1, and significantly inhibited the proliferation of colon cancer HT29 cells [158].

In addition, the Nrf2/HO-1 axis also shows the ability of anti-tumor metastasis. Topiramate inhibits angiogenesis and exerts anti-metastatic effects by inducing an increase in Nrf2/HO-1 levels and the down-regulation of VEGF and carbonic anhydrases II and IX [159]. MMP-2 and MMP-9 are two enzymes involved in tumor progression. Manuka honey inhibits the activity of antioxidant enzymes and the expression of Nrf-2 and HO-1 to augment 5-FU-induced oxidative stress. The anti-metastatic effect of 5-FU was affected by decreasing the migration capacity, inhibiting the expression of MMP-2 and MMP-9, and increasing N-calmodulin and E-calmodulin [160]. Therefore, the activation of the Nrf2/HO-1 axis may have potential inhibitory effects on colon cancer proliferation and metastasis, highlighting its prospective significance as a therapeutic target for metastatic colon cancer.

The activation of the Nrf2/HO-1 axis plays a crucial role in the chemotherapy resistance of colon cancer [161]. 5-Fluorouracil (5-FU) is the most commonly used chemotherapy drug for colon cancer, but the development of 5-FU resistance has greatly reduced its clinical efficacy. Tumor stem cells are a subgroup of colon cancer cells, which can counteract 5-FU-induced oxidative damage in colon cancer cells by generating an adaptive cellular response to ROS. This mechanism is closely related to Nrf2 activation, which can lead to the upregulation of antioxidant enzymes and increase the drug resistance of cancer cells to 5-FU [162,163]. A study showed that the combined use of *Quercetin* and 5-FU weakens the level of Nrf2/HO-1 pathway-related markers in colon cancer cells and 5-FU-resistant colon cancer cells, so that quercetin reverses 5-FU resistance in colon cancer cells by regulating the NRF2/HO-1 pathway [164]. Similarly, Waghela et al. proposed that the expression of HIF-1α, Nrf-2, and HO-1 in HCT-116/R cells was high, and they were highly resistant to 5-FU. Compared with 5-FU alone, 5-FU combined with ML385 (a specific Nrf2 inhibitor) enhanced the 5-FU-mediated apoptotic death of HCT-116/R cells [161]. Kang et al. examined the epigenetic changes related to Nrf2 induction in the human CRC cell line (SNUC5) resistant to 5-FU. They found that the protein expression and activity of activated Nrf2 and HO-1 in SNUC5/5-FUR cells increased, and that the knockdown of Nrf2 or HO-1 significantly inhibited cancer cell survival and tumor growth in vitro and in vivo, leading to enhanced 5-FU sensitivity. Therefore, the mechanism of CRC acquiring 5-FU resistance involves upregulating the expression of Nrf2 and HO-1 through the epigenetic modification of DNA demethylation [139,140]. In addition, Luo et al. investigated cisplatin resistance by using the cisplatin-resistant cell line HCT116R. The results showed that the siRNA targeting of MIR4435-2HG in the cisplatin-resistant cell line HCT116R inhibited the levels of Nrf2 and HO-1 mRNA, so that MIR4435-2HG may promote the development of cisplatin resistance through the Nrf2/HO-1 pathway [165]. Oxaliplatin resistance affects the efficacy of treatment of colorectal cancer. Luteolin inhibited the Nrf2/HO-1 pathway in oxaliplatin-resistant cell lines in a dose-dependent manner and restored the sensitivity of oxaliplatin-resistant cell lines to chemotherapy drugs, but it had no obvious effect on Nrf2^-^/^-^ mice [166]. The above research suggests that the activation of the Nrf2/HO-1 axis is one of the reasons for the drug resistance of colon cancer chemotherapy. The inhibition of the activation of the Nrf2/HO-1 axis has an important impact on the development of the chemotherapy resistance of colon cancer.

The activation of ferroptosis can effectively prevent tumor progression and enhance the efficacy of targeted therapy and chemotherapy [167]. Ferroptosis inducers have been widely studied as a promising new method to fight drug-resistant cancer [168]. The activation of the Nrf2/HO-1 signaling pathway may play a key role in inducing ferroptosis [169]. Wei et al. found that *Tagitinin C* inhibited the growth of colorectal cancer cells, including HCT116 cells, and induced ROS accumulation. ROS accumulation and Nrf2/HO-1 specific activation led to iron cell death [169]. The results showed that *hesperidin* upregulated the levels of ROS, lipid peroxidation, MDA, and Fe^2+^ in HCT116 cells and AOM/DSS-induced CRC mice, as well as significantly increased the levels of key ferroptosis genes, such as COX2 and ASCL4 proteins, and down-regulated FTH1. Iron accumulation and lipotoxicity were alleviated in HCT116 cells after HO-1 knockdown, the condition that is precisely due to the specific activation of the Nrf2/HO-1 pathway. Therefore, *heptapeptin* may induce ferroptosis in HCT116 cells by activating the Nrf2/HO-1 signaling pathway [170]. On the contrary, by eliminating lipid oxidation, the activation of the Nrf2/HO-1 axis may inhibit ferroptosis [171]. Cetuximab was found to inhibit the Nrf2/HO-1 signaling pathway in KRAS mutant CRC, promoting RSL3-induced ferroptosis and overcoming drug resistance in KRAS-mutant CRC [71]. Wu et al. studied that *ginsenoside Rh3* inhibited the Stat3/p53/NRF2 axis, promoting the depletion of GSH and the accumulation of iron, lipid ROS, and MDA that ultimately led to ferroptosis in CRC tumor cells [172]. The above research shows that the Nrf2/HO-1 axis has a bidirectional regulation function in the ferroptosis of CRC, which can be used as a pharmacological target of colorectal cancer and provide a new direction for the choice of future treatment strategies.

## 5. Phytochemicals Targeting the Nrf2/HO-1 Signaling Pathway in the Treatment of IBD

As an important regulatory factor in antioxidant reactions, Nrf2 is activated and released under stress to induce the expression of HO-1 to maintain intracellular redox homeostasis. HO-1 is regulated by Nrf2, which converts hemoglobin into CO and Fe^2+^, and reduces the above products to bilirubin, exerting antioxidant, anti-inflammatory, anti-apoptotic, and antithrombotic effects [173]. Previous studies have shown that promoting Nrf2/HO-1 pathway signaling enhances the antioxidation and anti-inflammatory abilities of the body, protects intestinal epithelial cells from damage, and maintains the integrity of the intestinal mucosal barrier [174]. Recently, a lot of studies have shown that Nrf2/HO-1 is the key crosstalk path of ferroptosis-influenced signaling pathways in IBD. To sum up, Nrf2/HO-1 may become a new hotspot for therapeutic targeting in IBD.

In clinical practice, various phytochemicals have been used to treat IBD. Recently, many studies have focused on discovering the potential of natural compounds in the treatment of IBD. A variety of natural molecules and extracts has been shown to treat IBD by activating the Nrf2/HO-1 signaling pathway. We summarized the specific mechanisms of treating IBD by activating the Nrf2/HO-1 signal pathway through natural products (Table 1), natural extracts (Table 2), and chemical drugs (Table 3). The literature showed that Nrf2/HO-1 exerts the therapeutic effect of IBD mainly through three mechanisms: Firstly, crosstalking multiple pathways, such as NF-κB and NLRP3 inflammatory vesicles, modulating inflammatory mediators, and inducing antioxidant enzyme production; secondly, regulating intestinal microbiota, promoting intestinal epithelial tight junctions, and maintaining the integrity of the intestinal mucosal barrier; and thirdly, targeting programmed cell death in IBD, including cellular pyroptosis, autophagy, and ferroptosis, by mediating the death of intestinal epithelial cells and immune cells. However, further studies are essential to elucidate the complex role of Nrf2/HO-1 and develop new drugs that modulate the Nrf2/HO-1 pathway and enhance its defense effects.

## 6. Conclusions and Opinions

The Nrf2/HO-1 signaling pathway plays a key role in the development of the intestine and the maintenance of its normal function. Currently, the role of the Nrf2/HO-1 signaling pathway in the pathogenesis and complications of IBD has been thoroughly studied, and the importance of this pathway for the treatment of IBD has been largely clarified. In this review, we described the structure and function of Nrf2/HO-1 and its role in the intestine, while elucidating the key role of the Nrf2/HO-1 signaling pathway in IBD and its complications. Finally, we summarized the potential of phytochemicals to treat IBD through this pathway.

However, the current studies described in our review mainly focus on animal and cellular models that lack the support of clinical trial data, which is some distance from a clinical translation. In the future, a more nuanced understanding of the exact mechanisms involved in the Nrf2/HO-1 pathway is the way forward for the treatment of IBD. This will help us to understand the directionality of the disease process, including potential episodes or complications of IBD. The goal for the future is to maintain an optimal balance of Nrf2/HO-1 axis activity—a key challenge because both the deficiency and over-activation of Nrf2 and HO-1 exacerbate disease. In addition, the discovery of novel natural and synthetic compounds that interact with the Nrf2/HO-1 pathway offers exciting prospects for the development of new therapies. These compounds may have potent antioxidant and anti-inflammatory properties, and their potential for safe and effective IBD management is promising.

In conclusion, studies of the Nrf2/HO-1 pathway and its complex interactions with IBD have revealed the frontiers of therapeutic strategies. However, the pathways for the molecules upstream and downstream of this pathway are not fully understood, and human evidence to support therapeutic interventions remains scarce. The challenge is to determine how to optimize the action of this mechanism, to understand their long-term effects, and to explore the new opportunities they may present. By conducting these studies, we are moving toward a common goal: to elucidate the mechanisms of the effects of the Nrf2/HO-1 pathway on IBD and the complex role of potential drugs targeting Nrf2/HO-1 in the prevention of IBD and its chronic complications.

## Figures and Tables

**Table 1:** Natural products modulating the Nrf2/HO-1 signaling pathway against IBD.

Categories	Natural Product	Experiment Models	Effective Dose	Associated Phenotypes	Potential Mechanism	PMID
Single drug	*Licorice*	DSS induced in mouse model	160 mg/kg	IL-1β↓, IL-6↓, IL-17↓, TNF-α↓, SOD↑, GPX↑, MDA↓, Nrf2↑, PINK1↑, Parkin↑, HO-1↑, P62↑, LC3↑	Nrf2/PINK1 pathway	36705402
	*Licorice*	DSS induced in mouse model	160 mg/kg	MDA↓, IL-1β↓, IL-6↓, TNF-α↓, SOD↑, GSH-PX↑, IL-10↑, Nrf2↑, PINK1↑, Parkin↑, HO-1↑, ZO-1↑, Occludin↑, P62↑	Nrf2/PINK1 signaling pathway	38176667
	*Crocus sativus*	TNBS and DSS induced in mouse model	20 mg/kg	Colon length↑, HO-1↑, GPX-2↑, TNF-α↓, IFN-γ↓	Ahr/Nrf2 pathway	34275090
	*Tetrastigma hemsleyanum leaves*	DSS induced in mouse model	-	Colon length↑, ZO-1↑, Occludin↑, Claudin-1↑, IL-1β↓, IL-6↓, TNF-α↓, SOD↑, CAT↑, HO-1↑, NQO1↑, GCLC↑, MPO↓, MDA↓, Nrf2↑	Keap1/Nrf2 signaling pathway	34747421
	*Ficus pandurate Hance*	DSS induced in mouse model	480 mg/kg	MPO↓, ZO-1↑, Occludin↑, SOD↑, GSH-Px↑, NRF2↑, HO-1↑, NQO1↑, MDA↓, Keap1↓, NOX2↓	Nrf2 pathway	34966476
*Polyphenols*	*Paeoniflorin*	LPS induced in Caco-2 cell	100 μm	COX-2↓, INOS↓, TNF-α↓, IL-6↓, Occludin↑, ZO-1↑, Claudin-5↑	Nrf2/HO-1 signaling pathway	31473900
	*Puerarin*	DSS induced in mouse model	50 mg/kg	MPO↓, TNF-α↓, IL-1β↓, IL-6↓, IFN-r↓, Nrf2↑, HO-1↑, NQO1↑, MDA↓, CAT↑, GSH↑, SOD↑, ZO-1↑, Occludin↑, Claudin-1↑, COX-2↓, INOS↓	Nrf2 pathway	31981944
	*Honokiol*	DSS induced in mouse model	40 mg/kg	IL-1β↓, IL-6↓, TNF-α↓, INOS↓, COX-2↓, ZO-1↑, Occludin↑, Claudin-1↑	Nrf2/HO-1 signaling pathway	36578522
	*Myristicin*	AA induced in rat model	150 mg/kg	MPO↓, TNF-α↓, IL-1β↓, COX-2↓, IL-10↑, Nrf2↑, HO-1↑	Nrf2/HO-1 signaling pathway	36403646
	*Dieckol*	DSS induced in mouse model	15 mg/kg	TNF-α↓, IL-1β↓, MDA↓, MPO↓, Nrf2↑, HO-1↑, Colon length↑	Nrf2/HO-1 signaling pathway	33331035
	*Resveratrol*	DSS induced in mouse model	100 mg/kg	IL-6↓, IL-1β↓, TNF-α↓, IL-10↑, ZO-1↑, Occludin↑, Nrf2↑, HO-1↑	Nrf2/HO-1 pathway	38488031
	*coumaric acid and syringic acid*	AA induced in rat model	150 mg/kg, 50 mg/kg	TNF-α↓, IL-1β↓, HO-1↑, Nrf2↑, NQO1↑	Nrf2/HO-1 pathway	37182450
	*Luteolin*	DSS induced in mouse model	50 mg/kg	Colon length↑, INOS↓, TNF-α↓, IL-6↓, MDA↓, SOD↑, CAT↑, Nrf2↑, HO-1↑, NQO1↑	Nrf2/HO-1 pathway	27569028
	*Rosmarinic Acid-Loaded Nanovesicles*	DSS induced in mouse model	20 mg/kg	MPO↓, TNF-α↓, IL-1β↓, NLRP3↓, ASC↓, caspase-1↓, Nrf2↑, HO-1↑	NLRP3 pathway	33530569
	*Synthetic Imine Resveratrol Analog 2-Methoxyl-3,6-Dihydroxyl-IRA*	DSS induced in mouse model	200 mg/kg	Colon length↑, IL-6↓, TNF-α↓, HO-1↑, NLRP3↑	Nrf2 pathway	31885813
	*Oligonol*	DSS induced in mouse model	100 mg/kg	Colon length↑, IL-1β↓, IL-6↓, TNF-α↓, HO-1↑, NQO-1↑	Nrf2 pathway	30149369
	*Licochalcone A*	DSS induced in mouse model	80 mg/kg	Colon length↑, MPO↓, TNF-α↓, IL-1β↓, IL-6↓, COX-2↓, GSH↑, SOD↑, NO↓, Nrf2↑, HO-1↑, GCL↑, Keap-1↓	Nrf2 pathway	29710547
	*Cyanidin-3-glucoside and resveratrol*	Cytokine induced in HT-29 intestinal cells	25 μM	Nrf2↑, HO-1↑, GSH/GSSG↑	Nrf2 pathway	27818126
	*Carnosic acid*	DSS induced in mouse model	100 mg/kg	Colon length↑, TNF-α↓, IL-6↓, IFN-γ↓, IL-1β↓, IL-18↓, HO-1↑, GPX2↑, SOD↑, Nrf2↑, GSH↑, SOD↑, MDA↓, INOS↓	Keap1/Nrf2 pathway	28887507
	*Sesamin*	DSS induced in mouse model	100 mg/kg	Colon length↑, IL-6↓, IL-1β↓, TNF-α↓, Nrf2↑, GSH↑, SOD↑, MDA↓, HO-1↑	Nrf2 pathway	31534619
*Polysaccharide*	*Astragalus polysaccharide*	DSS induced in mouse model	300 mg/kg	Histological damage↓, IFN-γ↓, IL-6↓, TNF-α↓, IL-1β↓, MDA↓, GSH↑	Nrf2/HO-1 pathway	34562468
	*Dandelion polysaccharide*	DSS induced in mouse model	300 mg/kg	Nrf2↑, HO-1↑, NQO-1↑, GSH↑, MDA↓, MPO↓, IL-1β↓, IL-6↓, TNF-α↓, INOS↓, Occludin↑, ZO-1↑	Nrf2 pathway	37893693
	*Polysaccharides from garlic*	DSS induced in mouse model	300 mg/kg	Colon length↑, INOS↓, COX2↓, ZO-1↑, Occludin↑, MUC2↑, IL-1β↓, IL18↓, MDA↓, Keap-1↓, GPX4↑, SOD↑, HO-1↑, NQO1↑, Nrf2↑	Nrf2 pathway	36017235
	*Aloe polysaccharides*	DSS induced in mouse model	100 mg/kg	Colon length↑, GSH↑, CAT↑,SOD↑, MPO↓, IFN-γ↓, MDA↓, TNF-α↓, IFN-γ↓, IL-1β↓, IL-6↓, IL-8↓, IL-17↓, ZO-1↑, Occludin↑, Nrf2↑, HO-I↑, NQO-1↑, IL-10↑, ZO-1↑, Claudin-1↑	Nrf2/HO-1 signaling pathway	34229016
	*Dandelion polysaccharide*	DSS induced in mouse model	300 mg/kg	IL-1β↓, IL-6↓, TNF-α↓, INOS↓, ZO-1↑, Occludin↑, MDA↓, MPO↓	Nrf2 pathway	37893693
	*Fucoidan*	AA induced in rat model	150 mg/kg	Colon length↑, IL-22↑, Nrf2↑, HO-1↑	Nrf2 pathway	35870906
*Terpenoids*	*Geniposide*	DSS induced in mouse model	60 mg/kg	Colon length↑, Histopathologic scores↓, ZO-1↑, Occludin↑, IL-1β↓, IL-6↓, TNF-α↓, MDA↓, NQO1↑, HO-1↑	Nrf2/ARE signaling pathway	37187359
	*Melianodiol*	DSS induced in mouse model	200 mg/kg	IL-10↑, IL-1β↓, TNF-α↓, MDA↓, NO↓, GSH↑, SOD↑, Nrf2↑, Keap-1↓, HO-1↑	Nrf2 signaling pathway	36312760
	*Ruscogenin*	TNBS induced in mouse model	2 mg/mouse	TNF-α↓, IFN-γ↓, Nrf2↑, NQO1↑	Nrf2/HO-1 signaling pathway	35308175
	*Crocin*	AA induced in rat model	20 mg/kg	Weight/length index↓, SOD↑, GSH↑, TAC↑, CAT↑, MDA↓, TNF-α↓, Nrf2↑, HO-1↑	Nrf2/HO-1 signaling pathway	30530041
	*Ginsenoside Rg1*	DSS induced in mouse model	200 mg/kg	Colon length↑, IL-1β↓, IL-6↓, TNF-α↓, SOD↑, MDA↓, MPO↓	Nrf2/HO-1/NF-kB pathway	38215064
	*Triptolide*	DSS induced in mouse model	0.02 mg/kg	Colon length↑, Claudin-1↑, Occludin↑, IL-1β↓, IL-6↓, ROS↓	NRF2/HO-1 signaling pathway	33240279
	*Masticadienonic acid*	DSS induced in mouse model	100 mg/kg	TNF-α↓, IL-1β↓, IL-6↓, MAPK↓, NF-κB↓, ZO-1↑, Occludin↑	Nrf2/HO-1 and MAPK/NF-κB signaling pathway	36403513
	*Geniposide*	DSS induced in mouse model	40 mg/kg	IL-6↓, IL-1β↓, TNF-α↓, MDA↓, SOD↑, MPO↓, Nrf2↑, HO-1↑, p-NF-κBp65↓, p-IκBα↓	Nrf2/HO-1/NF-κB pathway	32787366
	*Huzhangoside C*	DSS induced in mouse model	-	Colon length↑, INOS↓, MDA↓, NO↓, Nrf2↑, SOD↑, GSH↑	Nrf2 pathway	38156815
	*Nerolidol*	DSS induced in mouse model	150 mg/kg	MPO↓, IL-6↓, IL-1β↓, TNF-α↓, COX-2↓, INOS↓, Keap-1↓, Nrf2↑, SOD↑, HO-1↑	Nrf2 pathway	32650602
	*Tussilagone*	DSS induced in mouse model	2.5 mg/kg	Colon length↑, MPO↓, INOS↓, COX-2↓, TNF-α↓, IL-6↓, Nrf2↑, HO-1↑	Nrf2 pathway	30142311
	*Asperuloside*	DSS induced in mouse model	500 μg/kg	Colon length↑, Histopathological score↓, MPO↓, SOD↑, GSH-Px↑, MDA↓, Nrf2↑, HO-1↑, NQO1↑, TNF-α↓, IL-6↓, IL-10↑	Nrf2/HO-1 pathway	33974900
	*D-Pinitol*	DSS induced in mouse model	40 mg/kg	Colon length↑, HO-1↑, NQO1↑, GSH↑, SOD↑, CAT↑, MPO↓, MDA↓, TNF-α↓, IFN-γ↓, IL-6↓, IL-1β↓, INOS↓, COX-2↓, IL-10↑	Nrf2/ARE pathway	33625409
	*Sericic acid*	DSS induced in mouse model	50 mg/kg	Colon length↑, NO↓, TNF-α↓, IL-6↓, IL-1β↓, MDA↓, SOD↑, Nrf2↑, HO-1↑	Nrf2 pathway	36173058
	*Loganic acid*	DSS induced in mouse model	30 mg/kg	Colon length↑, MDA↓, NO↓, MPO↓, GSH↑, IL-1β↓, IL-6↓, TNF-α↓, TLR4↓, NF-κB↓, MPO↓, IFN-γ↓, Nrf2↑, HO-1↑, SOD↑	TLR4/NF-κB and SIRT1/Nrf2 pathway	37421777
	*Toosendanin*	DSS induced in mouse model	1 mg/kg	Colon length↑, MPO↓, TNF-α↓, IL-1β↓, IL-6↓, SOD↑, GSH↑, MDA↓, Nrf2↑, HO-1↑, ZO-1↑, Occludin↑	Nrf2/HO-1 pathway	31520988
	*RH-F/C-NPs*	DSS induced in mouse model	25 mg/kg	Colon length↑, MPO↓, TNF-α↓, IL-1β↓, IL-6↓, TLR4↓, NF-κB↓, GSH-PX↑, SOD↑, MDA↓, Nrf2↑, HO-1↑, ZO-1↑, Claudin-1↑, Occludin↑	TLR4/NF-κB and Nrf2/HO-1 signaling pathway	36326017
	*American ginseng, panaxynol, hexane fraction*	DSS induced in mouse model	75 mg/kg, 1 mg/kg, 75 mg/kg	COX2↓, HO-1↑	Nrf2 pathway	32575883
*Alkaloid*	*Oxyberberine*	TNBS induced in rat model	50 mg/kg	IL-4↑,TNF-α↓, IL-2↓, IL-8↓,IL-22↓, HO-1↑, GCLM↑, GCLC↑, NQO-1↑, SOD↑, GSH↑, MDA↓, ROS↓, CAT↑, Keap1↓, Nrf2↑	Keap1/Nrf2/NF-κB pathway	37247589
	*Oleracein E*	TNBS induced in rat model	20 mg/kg	IL-6↓, IL-1β↓, TNF-α↓, CAT↑, MPO↓, ROS↓, ZO-1↑, Occludin↑, Claudin-2↑, Nrf2↑, HO-1↑	Nrf2/HO-1 pathway	37395238
	*Berberine*	AA induced in rat model	50 mg/kg	IL-1β↓, IL-6↓, TNF-α↓, MPO↓, GSH↑, SOD↑, CAT↑, GPx↑, MDA↓, NO↓, Nrf2↑, HO-1↑	Nrf2/HO-1 pathway	33061833
	*Sinomenine*	DSS induced in rat model	40 mg/kg	Colon length↑, NO↓, MPO↓, SOD↑, CAT↑, GPx↑, MDA↓, TNF-α↓, IL-1β↓, IL-6↓, IL-10↑, HO-1↑, Nrf2↑, COX-2↓, INOS↓	HO-1/Nrf2 pathway	38573363
	*Corynoline*	DSS induced in mouse model	30 mg/kg	Colon length↑, Histological scores↓, MPO↓, IL-1β↓, IL-6↓, TNF-α↓, SOD↑, CAT↑, ROS↓, Nrf2↑, HO-1↑, IκBα↓, NF-κB p65↓	Nrf2/HO-1/NF-κB pathway	35980837
	*8-Oxypalmatine*	DSS induced in mouse model	50 mg/kg	Colon length↑, Histological scores↓, MPO↓, TNF-α↓, IL-1β↓, IFN-γ↓, IL-6↓, SOD↑, GSH↑, CAT↑, GSH-Px↑, MDA↓, NLRP3↓, Nrf2↑, HO-1↑	Nrf2 signaling pathway	35779424
	*Leonurine*	DSS induced in mouse model	30 mg/kg	Colon length↑, TNF-α↓, IL-6↓, IL-1β↓, MDA↓, ROS↓, SOD↑, GSH↑, TLR4↓, p-NF-κB↓, Nrf2↑, HO-1↑	Nrf2/HO-1 and TLR4/NF-κB pathway	35253649
	*Sanguinarine*	NSAIDs-induced small intestinal inflammation in rat model	3.3 mg/kg	TDI↓, CMDI↓, LDH↓, ZO-1↑, TNF-α↓, IL-6↓, IL-1β↓, SOD↑, MDA↓, Nrf2↑, HO-1↑, Keap-1↓, P-p65↓	Nrf2/NF-κB pathways	36304153
*Flavonoid*	*Galangin*	DSS induced in mouse model	40 mg/kg	TNF-α↓, IL-6↓, IL-10↑, MPO↓, SOD↑, COX-2↓, INOS↓, Nrf2↑, HO-1↑, GST↑, GSH↑, SOD↑	Nrf2 pathway	31147743
	*Cardamonin*	TNBS and DSS induced in mouse model	60 mg/kg	Colon length↑, Nrf2↑, NQO1↑, Trx1↑, SOD↑, HO-1↑, MPO↓, IL-1β↓, TNF-α↓, IL-6↓	AhR/Nrf2/NQO1 pathway	30071202
	*Flavonoid isoliquiritigenin*	TNF-α-induced HT-29 cells	20 uM	Nrf2↑, HO-1↑, NQO1↑, IL-8↓, IL-1β↓, COX-2↓	Nrf2 pathway	28012970
	*LL202*	TNBS induced in mouse model	30 mg/kg	IL-1β↓, IL-6↓, TNF-α↓, SOD↑, GSH↑, TAC↑, MDA↓, Nrf2↑, HO-1↑	Nrf2/HO-1 pathway	30426485
	*Alpinetin*	DSS induced in mouse model	100 mg/kg	Colon length↑, MPO↓, Occludin↑, ZO-1↑, Claudin-2↑, MDA↓, SOD↑, Nrf2↑, HO-1↑	Nrf2/HO-1 signaling pathway	29661352
	*Diosmetin (3′,5,7-trihydroxy-4′-methoxy flavone)*	DSS induced in mouse model	50 mg/kg	Occludin↑, Claudin-1↑, ZO-1↑, IL-1β↓, IL-6↓, TNF-α↓, COX-2↓, GSH-Px↑, SOD↑, MDA↓, GSH↑, ROS↓, Nrf2↑, HO-1↑	Nrf2 pathway	34262136
	*Diosmin*	DSS induced in mouse model	200 mg/kg	Colon length↑, GSH↑, CAT↑, Nrf2↑, HO-1↑, MDA↓, NO↓, Mucin-2↑, ZO-1↑, Histopathological score↓, NF-κB↓, TNF-α↓, IL-6↓, Claudin-1↑, Occludin↑	NF-κB/Nrf2 pathway	38500992
	*Quercetin nanoparticles*	DSS induced in mouse model	20 mg/kg	GSH-Px↑, SOD↑, CAT↑, ROS↓, NO↓, MDA↓, IL-6↓, TNF-α↓, IFN-γ↓, IL-10↑, MUC-2↑, Occludin↑, Nrf2↑, HO-1↑, INOS↓, COX2↓	Nrf2 pathway	35884960
	*Hyperoside*	DSS induced in mouse model	120 mg/kg	Colon length↑, MDA↓, TNF-α↓, IL-6↓, COX-2↓, IL-10↑, MDA↓, Nrf2↑, HO-1↑, SOD↑	Nrf2 pathway	29162986
	*Sulforaphane*	AA induced in rat model	15 mg/kg	Colon length↑, Nrf2↑, HO-1↑, NO↓, MDA↓, GSH-Px↑, GSH↑	Nrf2 pathway	35754320
	*Genistein*	AA induced in rat model	25 mg/kg	Nrf2↑, HO-1↑, Caspase-3↑	Nrf2/HO-1 pathway	37084167
	*Diclofenac and eugenol hybrid*	DSS induced in mouse model	40 mg/kg	Colon length↑, p-NF-κB↓, NF-κB↓, HO-1↑, ROS↓, Nrf2↑, INOS↓	Nrf2/HO-1 and INOS/NF-κB signaling pathway	37517204
*Coumarins*	*Imperatorin*	TNBS induced in rat model	60 mg/kg	TNF-α↓, IL-6↓., Nrf2↑, ARE↑, HO-1↑	Nrf2/ARE/HO-1 pathway	33098052
	*Isofraxidin*	DSS induced in mouse model	80 mg/kg	Colon length↑, IL-8↓, TNF-α↓, MPO↓, ZO-1↑, IL-1β↓, IL18↓, ROS↓, Nrf2↑, HO-1↑, MDA↓, SOD↑	Nrf2 pathway	38280336

ARE, antioxidant response element; CAT, catalase; COX-2, cyclooxygenase-2; GCL, glutamate cysteine ligase; GCLM, glutamate–cysteine ligase modifier subunit; GCLC, glutamate–cysteine ligase catalytic subunit; GPx, glutathione peroxidase; IL, interleukin; INOS, inositol; IFN-γ, interferon-gamma; MDA, malondialdehyde; NF-κB, nuclear factor kappa B; MPO, myeloperoxidase; NO, nitric oxide; Nrf2, nuclear factor-E2-related factor 2; NQO1, NAD(P)H: quinone oxidoreductase; ROS, reactive oxygen species; SOD, superoxide dismutase; TNF, tumor necrosis factor; TLR4, toll-like receptor 4; ZO-1, zonula occludens-1. The “↑” and “↓” in the table indicate increase and decrease respectively.

**Table 2:** Natural extracts modulating the Nrf2/HO-1 signaling pathway against IBD.

Natural Extract	Experiment Models	Effective Dose	Associated Phenotypes	Potential Mechanism	PMID	
*Aucklandia lappa Decne extract*	DSS induced in mouse model	182 mg/kg	Colon length↑, TNF-α↓, IL-6↓, IL-1β↓	Nrf2-HO-1 signaling pathway	35623504	
*Lotus Leaf Extract*	LPS induced in mouse model	200 mg/kg	ZO-1↑, Occludin↑, Claudin-1↑, IL-1β↓, IL-6↓, TNF-α↓, Nrf2↑, HO-1↑	Nrf2/HO-1 signaling pathway	38526570	
*Grape seed proanthocyanidin extract*	DSS induced in mouse model	-	IL-1β↓, IL-6↓, TNF-α↓, NO↓, MDA↓, SOD↑, NF-κB↓, Keap-1↓, Nrf2↑, HO-1↑	NF-κB and Nrf2 pathway	35692132	
*Green pea hull*	DSS induced in mouse model	600 mg/kg	Colon length↑, MPO↓, Claudin-1↑, Occludin↑, ZO-1↑, MDA↓, SOD↑, CAT↑, TNF-α↓, IL-1β↓, IL-6↓, IL-10↑, Keap1↓, Nrf2↑, GCLC↑, HO-1↑, NQO1↑	Keap1-Nrf2-ARE signaling pathway	34829046	
*Moringa seed extract*	DSS induced in mouse model	150 mg/kg	Colon length↑, TNF-α↓, NO↓, MPO↓, IL-1β↓, IL-6↓, TNF-α↓, INOS↓, Claudin-1↑, ZO-1↑, NQO1↑, HO-1↑	Nrf2 Pathway	28922365	
*Extract of Rhus chinensis Mill. fruits*	DSS induced in mouse model	600 mg/kg	Colon length↑, MDA↓, MPO↓, TNF-α↓, IL-1β↓, IL-6↓, SOD↑, GSH↑, Occludin↑, ZO-1↑, Claudin-1↑, Nrf2↑, NQO1↑, HO-1↑, COX-2↓, INOS↓,	Nrf2 pathway	34494061	
*Bruguiera gymnorrhiza fruit*	DSS induced in mouse model	100 mg/kg	Colon length↑, MDA↓, TNF-α↓, IL-6↓, IL-1β↓, IFN-γ↓, IL-10↑, SOD↑, GSH↑, Nrf2↑, HO-1↑, NQO1↑, Keap1↓	Keap1/Nrf2 pathway	32116661	
*ROS extract*	DSS induced in mouse model	500 mg/kg	Colon length↑, INOS↓, MPO↓, MDA↓, p-NF-κB↓, p-IKKα/β↓, Keap1↓, Nrf2↑, HO-1↑, SOD↑, TNF-α↓, IL-6↓, IL-1β↓	Nrf2/NF-κB pathway	35692958	
*Perilla frutescens extract*	DSS induced in mouse model	100 mg/kg	COX-2↓, INOS↓, HO-1↑, Nrf2↑, TNF-α↓, IL-10↑	Nrf2 pathway	28848431	
*Artemisia argyi extract*	DSS induced in mouse model	200 mg/kg	IL-6↓, IL-1β↓, TNF-α↓, p-IκBα↓, p-NF-κB↓, Cox2↓, Nrf2↑, HO-1↑, MPO↓, INOS↓	NF-κB/Nrf2 pathway	35277165	
*Maggot extract*	DSS induced in mouse model	1000 mg/kg	Colon length↑, pIκB↓, IL-6↓, IL-1β↓, NFκB p65↓, TNF-α↓, Nrf2↑, HO-1↑, TGF-β1↓, SMADs↓	TGF-β1/SMAD pathway	34456904	
*Iziphus spina-christi fruit extract*	AA induced in rat model	400 mg/kg	Colon length↑, NO↓, MPO↓, GSH↑, SOD↑, CAT↑, GPx↑, Nrf2↑, HO-1↑, IL-1β↓, INOS↓, TNF-α↓, COX-2↓	Nrf2/HO-1 pathway	29518435	
*Extract of Mesua Assamica (King & prain) Kosterm.*	DSS induced in mouse model	200 mg/kg	Colon length↑, NO↓, MDA↓, MPO↓, GSH↑, IL-1β↓, IL-6↓, TNF-α↓, Nrf2↑, HO-1↑, SOD↑,	HO-1/Nrf2/SIRT1 signaling pathway	36195303	
*24Z-masticadienonic acid*	DSS induced in mouse model	100 mg/kg	Colon length↑, IL-1β↓, IL-6↓, TNF-α↓, Occludin↑, ZO-1↑, Nrf2↑, HO-1↑, NFκB p65↓, pIκB↓	Nrf2/HO-1 and NF-κB pathway	36403513	
*Gingerenone A*	DSS induced in mouse model	20 mg/kg	IL-1β↓, IL-6↓, TNF-α↓, MDA↓, GSH↑, Nrf2↑, HO-1↑, NQO1↑, GPX4↑	Nrf2–GPX4 signaling pathway	36135333	

ARE, antioxidant response element; CAT, catalase; COX-2, cyclooxygenase-2; GCLC, glutamate–cysteine ligase catalytic subunit; GPX4, glutathione peroxidase 4; GSH-PX, glutathione peroxidase; IL, interleukin; INOS, inositol; IFN-γ, interferon-gamma; MDA, malondialdehyde; NF-κB, nuclear factor kappa B; MPO, myeloperoxidase; NO, nitric oxide; Nrf2, nuclear factor-E2-related factor 2; NQO1, NAD(P)H: quinone oxidoreductase; ROS, reactive oxygen species; SOD, superoxide dismutase; TNF, tumor necrosis factor; ZO-1, zonula occludens-1. The “↑” and “↓” in the table indicate increase and decrease respectively.

**Table 3:** Chemosynthetic activators modulating the Nrf2/HO-1 signaling pathway against IBD.

Natural Extract	Experiment Models	Effective Dose	Associated Phenotypes	Potential Mechanism	PMID
Linagliptin	TNBS induced in rat model	1.5 mg/kg	IL-6↓, TNF-α↓, MPO↓, IL-10↑, IL-6↓, TNF-α↓, IL-10↑, GSH↑, GPx↑, TAC↑, Nrf2↑, HO-1↑	Nrf2/HO-1 pathway	33667522
Carbocisteine	AA induced in rat model	500 mg/kg	Histopathological score↓, TAC↑, HO-1↑, MPO↓, IL-6↓, TNF-α↓, IL-10↑, TLR4↓, Nrf2↑, NF-κB p-65↓	Nrf2/HO-1 and NFκB pathway	35754464
Olmesartan	AA induced in rat model	10 mg/kg	Weight/length ratio↓, IL-6↓, TNF-α↓, IL-1β↓, TGF-β↓, IL-10↑, Nrf2↑, HO-1↑, NFĸB p65↓, MPO↓, TAC↑, SOD↑, GSH↑, CAT↑, MDA↓, IL-10↑	NFκB and Nrf2/HO-1 pathway	30594690
Nadroparin sodium	AA induced in rat model	500 units/kg	NF-κB↓, AP-1↓, COX-2↓, IL-6↓, TNF-α↓, HO-1↑, Nrf2↑, MDA↓	Nrf2/HO-1 and NF-κB pathway	22350949
Levetiracetam	AA induced in mouse model	100 mg/kg	Colon length↑, TNF-α↓, IL-6↓, IL-1β↓, IFN-γ↓, IL-10↑, TGF-β↑, INOS↓, NO↓, GSH↑, SOD↑, CAT↑, MDA↓, MPO↓, Nrf2↑, HO-1↑, p-NF-κB↓	Nrf2/HO-1 and NF-κB pathway	37068340
Ambroxol	AA induced in rat model	200 mg/kg	Weight/length ratio↓, Nrf2↑, HO-1↑, IL-6↓, TNF-α↓, IL-10↑, CAT↑, TAC↑, MPO↓, IL-6↓, TNF-α↓, IL-10↑, IκBα↓, p65↓	NF-κB/Nrf2 pathway	35947115
Vildagliptin	AA induced in rat model	10 mg/kg	Nrf2↑, HO-1↑, NQO1↑, TNF-α↓, NF-κB↓, IL-1β↓, PI3K↓, Akt↓	PI3K/Akt/NFκB and Nrf2 signaling pathway	33434756
Dapagliflozin	TNBS induced in rat model	5 mg/kg	Weight/length ratio↓, TNF-α↓, MPO↓, IL-10↑, GSH↑, GPx↑, Nrf2↑, HO-1↑	Nrf2/HO-1 pathway	33412153
Sodium butyrate	DSS induced in mouse model	10 mg/kg	Colon length↑, MPO↓, MDA↓, GSH↑, COX-2↓, Nrf2↑, HO-1↑, IL-6↓, TNF-α↓, NF-κB↓, IL-1β↓, IL-18↓, NF-κB p65↓, NLRP3↓	NF-κB/NLRP3 and COX-2/Nrf2/HO-1 pathway	36867295
N-benzyl-N-methyldecan-1-amine; decyl-(4-methoxy-benzyl)-methyl-amine	DNBS induced in rat model	0.4 mg/kg	TNF-α↓, IL-1β↓, IL-6↓, HO1↑, Nrf2↑, MPO↓	Nrf2 pathway	37153778
NPs-PEG-FA/6-shogaol	DSS induced in mouse model	15 mg/kg	MPO↓, TNF-α↓, IL-6↓, IL-1β↓, INOS↓, Nrf2↑, HO-1↑	Nrf2 pathway	28961808
PEA/Polydatin	DNBS induced in rat model	10 mg/kg	MPO↓, MDA↓, IL-1β↓, TNF-α↓, NF-κB p65↓, IκB-α↑, INOS↓, SIRT-1↑, HO-1↑, Nrf2↑	NF-κB/SIRT1/Nrf2 pathway	33809584
Mo3Se4 nanoflakes	DSS induced in mouse model	100 mg/kg	Histopathological scores↓, MPO↓, ROS↓, MDA↓, CAT↑, GPx↑, SOD↑, GSH↑, Keap1↓, Nrf2↑, NQO1↑, HO-1↑, IL-1β↓, IL-6↓, TNF-α↓, IFN-β↓, TLR4↓, p-NF-κB↓, IκBα↑, MUC-2↑, Claudin-1↑, Occludin↑, ZO-1↑	Nrf2-Keap1 and TLR4/NF-кB pathway	35985164
I.e. diselenide-bridged hyaluronic acid nanogel	DSS and TNBS induced in mouse model	50 mg/kg	Colon length↑, MPO↓, MDA↓, SOD↑, HO-1↑, Nrf2↑, INOS↓, IL-6↓, TNF-a↓, IL-1β↓	Nrf2/HO-1 pathway	35861614
FA-97	DSS induced in mouse model	10 mg/kg	Colon length↑, MPO↓, INOS↓, IL-1β↓, IL-6↓, TNF-α↓, IL-12↓, IL-17↓, MDA↓, ROS↓, HO-1↑, NQO-1↑, Nrf2↑	Nrf2/HO-1 pathway	31969881
Mesalazine	TNBS induced in rat model	30 mg/kg	Nrf2↑, HO-1↑, MPO↓, COX-2↓, INOS↓	Nrf2-HO-1 pathway	28473247
Telmisartan	DSS induced in rat model	7 mg/kg	IL-1β↓, IL-6↓, TNF-α↓, Nrf2↑, HO-1↑, IL-10↑	NF-κB pathway	31326516
Coenzyme Q10	AA induced in rat model	100 mg/kg	Colon weight/colon length ratio↓, GSH↑, SOD↑, CAT↑, Nrf2↑, HO-1↑, MDA↓, TNF-α↓, Caspase-3↓	Nrf2/HO-1 pathway	28050757
Spermidine	DSS and TNBS induced in mouse model	20 mM	TNF-α↓, IL-6↓, Occludin↑, Claudin-1↑, Claudin-3↑, Nrf2↑, HO1↑, NQO1↑, SOD↑	AhR-Nrf2 pathway	37104918
Miconazole	AA induced in rat model	40 mg/kg	Histopathological score↓, MDA↓, SOD↑, GSH↑, IL-6↓, TNF-α↓, Nrf2↑, HO-1↑, IL-10↑, INOS↓, COX2↑, Caspase-3↓	Nrf2/HO-1 signaling pathway	35205169
Oat peptides	DSS induced in mouse model	500 mg/kg	IL-6↓, IL-1β↓,TNF-α↓, MDA↓, SOD↑, ZO-1↑, Occludin↑, Claudin-1↑, Nrf2↑, Keap1↓, NQO1↑, HO-1↑	Keap1-Nrf2 pathway	38140314
Dimethyl fumarate	DSS induced in mouse model	60 mg/kg	Colon length↑, MPO↓, INOS↓, IL-1β↓, IL-6↓, TNF-α↓, Nrf2↑, HO-1↑, NQO1↑	Nrf2/ARE pathway	27184504

CAT, catalase; COX-2, cyclooxygenase-2; GCLC, glutamate–cysteine ligase catalytic subunit; GPX4, glutathione peroxidase 4; GSH-PX, glutathione peroxidase; IL, interleukin; INOS, inositol; IFN-γ, interferon-gamma; MDA, malondialdehyde; NF-κB, nuclear factor kappa B; MPO, myeloperoxidase; MUC-2, mucin-2; NO, nitric oxide; Nrf2, nuclear factor-E2-related factor 2; NQO1, NAD(P)H: quinone oxidoreductase; ROS, reactive oxygen species; SOD, superoxide dismutase; TAC, total antioxidant capacity; TGF-β, transforming growth factor-beta; TLR4, toll-like receptor 4; TNF, tumor necrosis factor; ZO-1, zonula occludens-1. The “↑” and “↓” in the table indicate increase and decrease respectively.

## Data Availability

The data are contained in the article.
